# Social Restrictions versus Testing Campaigns in the COVID-19 Crisis: A Predictive Model Based on the Spanish Case

**DOI:** 10.3390/v13050917

**Published:** 2021-05-15

**Authors:** Francisco Javier Candel, Elisabet Viayna, Daniel Callejo, Raul Ramos, Jesús San-Roman-Montero, Pablo Barreiro, María del Mar Carretero, Adam Kolipiński, Jesus Canora, Antonio Zapatero, Michael Chris Runken

**Affiliations:** 1 Clinical Microbiology and Infectious Diseases, Hospital Clínico San Carlos, IdISSC and IML Health Institutes, Council of Public Health, Prof Martín Lagos, s/n, 28040 Madrid, Spain; franciscojavier.candel@salud.madrid.org; 2 Scientific & Medical Affairs, Global Health Economics and Outcomes Research, Grifols S.A., Av. Generalitat, 152 (SC3), Sant Cugat del Vallès, 08174 Barcelona, Spain; 3 Health Economics and Outcomes Research, IQVIA, Juan Esplandiú, 11, 28007 Madrid, Spain; daniel.callejovelasco@iqvia.com; 4 AQR-IREA, Deptment of Econometrics, Statistics and Applied Economics, Faculty of Economics and Business, University of Barcelona, Av. Diagonal, 690-696, 08034 Barcelona, Spain; rramos@ub.edu; 5 Department of Medical Specialties and Public Health, Rey Juan Carlos University, Avenida de Atenas s/n, Alcorcón, 28922 Madrid, Spain; jesus.sanroman@urjc.es; 6 Department of Infectious Diseases, Internal Medicine, Hospital La Paz, Council of Public Health, European University of Madrid, Paseo de la Castellana, 261, 28046 Madrid, Spain; pablo.barreiro@salud.madrid.org; 7 Public Health Laboratory, Council of Public Health, Calle Sierra de Alquife, 8, 28053 Madrid, Spain; maria.carretero@salud.madrid.org; 8 Software Development Stat Services, IQVIA Commercial sp. z o.o., Domaniewska 48, 02-672 Warsaw, Poland; adam.kolipinski@iqvia.com; 9 Health Council, Community of Madrid, Madrid, C/O’Donnell, 55, 4th Floor, 28009 Madrid, Spain; jesus.canora@salud.madrid.org (J.C.); antonio.zapatero@salud.madrid.org (A.Z.); 10 Scientific & Medical Affairs, Global Health Economics and Outcomes Research, Grifols SSNA, 79 TW Alexander Dr Bldg. 4101, Durham, NC 27713, USA; chris.runken@grifols.com

**Keywords:** SEIR, COVID-19, health policy, economic impact, molecular test

## Abstract

The global COVID-19 spread has forced countries to implement non-pharmacological interventions (NPI) (i.e., mobility restrictions and testing campaigns) to preserve health systems. Spain is one of the most severely impacted countries, both clinically and economically. In an effort to support policy decision-making, we aimed to assess the impacts of different NPI on COVID-19 epidemiology, healthcare costs and Gross Domestic Product (GDP). A modified Susceptible-Exposed-Infectious-Removed epidemiological model was created to simulate the pandemic evolution. Its output was used to populate an economic model to quantify healthcare costs and GDP variation through a regression model which correlates NPI and GDP change from 42 countries. Thirteen scenarios combining different NPI were consecutively simulated in the epidemiological and economic models. Both increased testing and stringency could reduce cases, hospitalizations and deaths. While policies based on increased testing rates lead to higher healthcare costs, increased stringency is correlated with greater GDP declines, with differences of up to 4.4% points. Increased test sensitivity may lead to a reduction of cases, hospitalizations and deaths and to the implementation of pooling techniques that can increase throughput testing capacity. Alternative strategies to control COVID-19 spread entail differing economic outcomes. Decision-makers may utilize this tool to identify the most suitable strategy considering epidemiological and economic outcomes.

## 1. Introduction

As of 18 March 2021, over 120 million COVID-19 confirmed cases have been reported globally, leading to over 2.6 million deaths [1]. Since the first COVID-19 cases reported in France and Germany at the end of January 2020 [2], the COVID-19 pandemic spread rapidly across Europe, forcing most governments to implement different approaches to social restrictions during Q2 2020 [3,4]. Those restrictions managed to reduce the spread of the pandemic, albeit at the expense of decreasing GDP 12.6% vs. Q1 2020 on average for the European Organization for Economic Co-operation and Development (OECD) countries [5].

With subsequent waves of COVID-19 cases currently taking place across European countries, and while vaccination rates are still low, policy makers are challenged with difficult decision-making that will impact not only the spread of the COVID-19 pandemic, but also the socioeconomic future of their countries. In order to achieve a balance between health and socioeconomic concerns for current and future COVID-19 waves, while vaccination immunity is not yet reached, interventions to control the pandemic need to be carefully assessed [6].

Spain was one of the first and most severely impacted countries by the COVID-19 pandemic in Europe [1,2,5,7]. Despite its health system being among the world’s best performing [8], the rapid spread of the first COVID-19 wave overwhelmed the health system, resulting in the government declaring the State of Emergency on 14 March 2020, and imposing one of the strictest lockdowns in Europe, in line with those imposed in Italy and France [3,4,9]. These restrictions managed to reduce the spread of COVID-19, but led to a 17.8% drop in GDP vs. Q1 2020, placing Spain in the top three OECD countries with the steepest GDP decline during Q2 2020 [5]. With an already fragile economy and exhausted health system, Spain has been facing waves of COVID-19 cases, leaving national and regional governments continually facing difficult policy decisions [7].

Massive testing strategies have been identified as the main alternative to social restrictions to manage the spread of COVID-19 [6,10]. From the very beginning of the COVID-19 pandemic, South Korea and Singapore employed testing rates of over 100 and 700 tests per case, respectively [4,10,11]. Spain, on the other hand, employed less than 10 tests per case during the first wave [4]. However, many SARS-CoV-2 tests initially showed high rates of false negatives, which have discouraged their use in massive testing strategies [12,13,14,15].

Several models have been published attempting to estimate the impact of different non-pharmacological interventions (NPI) (i.e., mobility restrictions and testing campaigns) on the pandemic and economic outcomes to better understand what measures may best help flatten the ‘new cases’ curve [12,16,17,18]. However, none of them used real data correlating the GDP variation with the NPI established or specifically exploring the impact of different strategies in Spain. In an effort to support decision-making processes, we aim to assess the epidemiological, direct healthcare costs, and GDP impacts of different social restrictions and testing strategies that may help control the COVID-19 pandemic based on the particular Spanish case.

## 2. Materials and Methods

Two models were utilized to estimate the clinical and economic impact of different NPI (lockdown/mobility restrictions and testing rates) on the COVID-19 pandemic in Spain. First, a dynamic modified Susceptible-Exposed-Infectious-Removed (SEIR) epidemiological model was developed to simulate the pandemic’s evolution and estimate the number of new cases, hospitalizations and deaths. Then, the results from the epidemiological model were used in a second model to estimate direct healthcare costs and change in GDP.

### 2.1. Epidemiological Model

**Figure 1:**
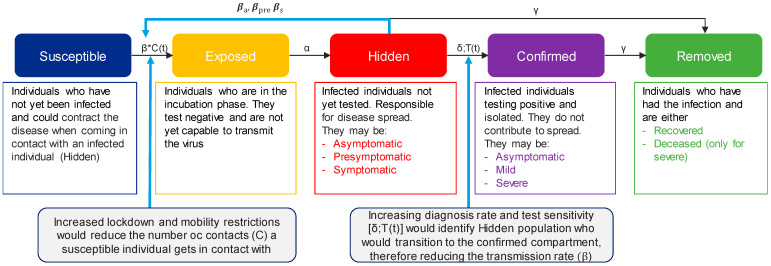
Diagram of the modified SEIR model. β represents the transmission rate and is adjusted based on whether the infectious individual is asymptomatic (βa = 0.002), pre-symptomatic (βs = 0.186) or symptomatic (βpre = 0.01); C(t) is the number of contacts at that moment t; α is the incubation time (α = 0.18); δ is the diagnosis rate and depends on the number of tests executed at that moment t [T(t)], the sensitivity of the test and the spread of the infection; γ is the recovery rate, which is adjusted based on whether the patient is mild (γ = 0.07) or severe (γ_R_ = 0.02) and the death rate for severe patients (γ_D_ = 0.009).

A dynamic, modified SEIR model was developed to simulate the number of new COVID-19 cases for the subsequent 90 days in Spain (Figure 1). Given the continuous changing environment of the pandemic, the 90-day timeframe was considered the most appropriate timeframe to provide estimates with lower error levels. The Spanish population was distributed into the following compartments based on their infection status: Susceptible (S), Exposed (E), Hidden infectious (H), Confirmed infectious (C) and Removed (R). Compared with the classic SEIR model, the infectious compartment was divided in two sub-compartments: hidden and confirmed. Hidden were those individuals who had not been tested and therefore were unaware they had the disease, making them the drivers for infection spread. Once tested, they transitioned to the confirmed infectious compartment, which consisted of patients who had tested positive and were assumed to isolate with no further disease transmission (Figure S1).

Susceptible individuals are those who have never been infected, but may become infected when coming in contact with an infectious individual. Their transition to exposed is driven by: the number of daily contacts of the susceptible individual, estimated by an age-adjusted contact matrix (Figure S2) [19]; age-adjusted herd immunity [20]; the sensitivity to become infected based on the age range (fitted data); the seasonality (fitted data); and the transmission rate (β) of the hidden patient, which is adjusted based on whether the infectious individual is asymptomatic (β_a_ = 0.002), pre-symptomatic (β_s_ = 0.186) or symptomatic (β_pre_ = 0.01).

Once a susceptible individual becomes infected, they transition to the exposed compartment, which corresponds to the incubation phase, so they cannot transmit the disease and would turn out negative if tested. New Exposed individuals in the moment *n* + 1 follow the formula:

E_(n+1)_ = (C_ij_ S_n_) (T)^T^ (h_n_)^T^ (Sens)^T^

where C_ij_ is the contact matrix between different age groups; S_n_ represents stringency measures; and T_n_ is the transition vector. It is estimated as follows:

1. The simulation is initiated at the hidden compartment at the moment n for each age group and type (asymptomatic, pre-symptomatic, symptomatic);
2. Then, the migration coefficient (constant number of pre-symptomatic individuals who are outside of the population) is added;
3. Then, multiplied by the respective transmission probability (β_a_ β_pre_ or β_s_) and by the seasonal adjustment.

h_n_ represents herd immunity for different age groups; and Sens represents the sensibility of the vector by age, (ranging from 0.1 for the 0–4 age group to 1.5 for the 80+ age group).

The transition from exposed to infectious is driven by the incubation time (α = 0.18), 5.6 days on average. After the incubation period, these individuals may become asymptomatic or pre-symptomatic, with different likelihood depending on age [21]. Individuals will remain pre-symptomatic for 2.7 days on average and then become symptomatic.

The transition from the hidden to the confirmed compartment is driven by the diagnosis rate (δ). Every day, a certain number of tests with a specific sensitivity are performed (which will depend on the scenario, as described in section “Scenarios Definition”). The tested population consists of a constant share of symptomatic individuals, a random share of hidden asymptomatic and pre-symptomatic, and non-infected individuals.

Finally, the removed compartment is divided into two sub-compartments: recovered and deceased. Hidden asymptomatic individuals who do not get tested, are assumed to transition to the recovered compartment 14 days later, on average. The transition from confirmed to removed depends on the severity of the symptoms (asymptomatic, mild or severe), which is adjusted by age. Asymptomatic and confirmed mild individuals will recover based on the recovery rate (γ = 0.07), but a share of confirmed mild will experience severe symptoms and require hospitalizations (μ = 0.037). Finally, a constant share of severe individuals will either transition to recovered (γ_R_ = 0.02) or deceased (γ_D_ = 0.009) (Tables S1 and S2).

In each compartment, the population was stratified by age using 5-year intervals. The model assumes the population remains constant from 31 January 2020, date of the first COVID-19 case in Spain, until the end of the simulation, assuming that no reinfection may occur. The model was populated and calibrated by adjusting the number of estimated confirmed patients to the number of actual confirmed patients as reported by Spanish Authorities, up to 11 September 2020 [22]. This dataset is published by the Spanish Government and is collected from the regional health authorities throughout the country on a daily basis using an electronic survey. Each positive patient has a unique identifier and, as part of the survey, authorities must report if this is a first infection or a re-infection. Therefore, no duplicates were expected. The SEIR demographic data were retrieved from the Spanish National Institute of Statistics.

The forecasting accuracy of the epidemiological model was assessed using the number of new confirmed cases, hospitalizations, and deaths reported by the Spanish Authorities on 19 October 2020. Our base case estimated 433,067 new confirmed cases for the first 30 days; this value was actually reached approximately 37 days after starting the simulation [22], therefore, proving a relatively accurate predictive capacity. However, the number of hospitalizations and deaths was overestimated with rates of 24.6% and 4.8%, respectively, considering the overall population. An adjustment was therefore made with a more conservative approach based upon the latest data reported by the Spanish Authorities placing the rate of hospitalization at 5.8% of total confirmed cases. By adjusting the rate of hospitalizations to 5.8%, the model predicted a death rate of 1.1%, well aligned with that reported by the Spanish Authorities. This adjustment in the hospitalization rate and the potential impact on the model results was evaluated in a deterministic one-way sensitivity analysis (Table S3).

### 2.2. Scenario Definitions

**Figure 2:**
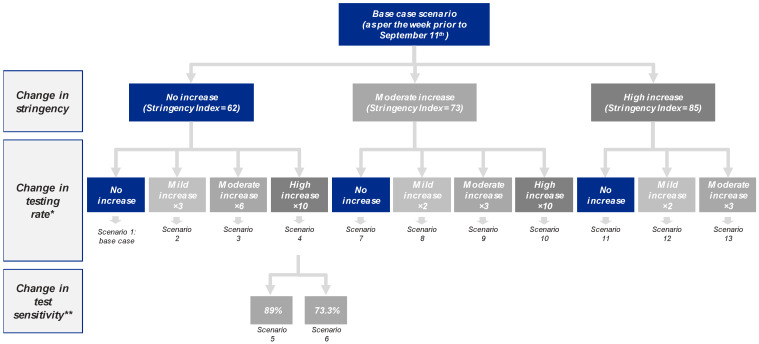
Scenarios considered by level of stringency, testing rate and test sensitivity. * Testing rate for scenario 1 (base case) is 10.6 tests per positive. ** All scenarios except 5 and 6 consider a sensitivity of 96%.

In order to explore the impact of different NPI, three variables were modified within the SEIR model, generating a total of 13 scenarios (Figure 2):

- Level of restrictions/lockdown (stringency) based on the Government Response Stringency Index, a composite measure on a 0-to-100 scale (100 being the strictest) based on nine response indicators including school/workplace closures and travel bans among others, which is fully described and available for download from the Our World in Data website [3,4];
- Number of molecular tests per case [4];
- Test sensitivity was assumed to be 96% (i.e., 4% false negative rate) in all scenarios (lower 95% confidence interval of the SARS-CoV-2 transcription-mediated amplification (TMA) Procleix^®^ test sensitivity) [23]. In two meta-analyses, other molecular tests had shown lower sensitivities [24,25]; conservatively, the sensitivity in the model was decreased to 89% and 73.3% to explore the relevance of this parameter [24,25].

The base case (scenario 1) was built considering a stringency of 62 [3,4], testing rate of 10.6 tests/positive [4], reflecting the situation reported the week prior to 11 September 2020 in Spain, and assuming 96% sensitivity [23].

A total of 10 alternative scenarios (2–4 and 7–13) were built adopting deviations from the base case in terms of stringency (moderate increase to 73 and high increase to 85, equivalent to the restrictions from April–May 2020 and March–April 2020 in Spain, respectively) and increased testing rate, aligned with that observed in other countries (with ×2, ×3, ×6 and ×10 increases) [4]. Finally, scenarios 5 and 6 explored the impact of test sensitivity, decreasing it from 96% to 89% and 73.3%, respectively [24,25].

For each scenario, new exposed cases, hospitalizations, Intensive Care Unit (ICU) admissions, and deaths were estimated in the epidemiological model and used as inputs for the economic model.

### 2.3. Economic Model

#### 2.3.1. Direct Healthcare Costs

Direct healthcare costs included testing, hospitalizations, ICU and primary care visits. In order to estimate the costs of testing, two different approaches were considered: individual testing and pooled testing. The pooling technique, broadly used in blood donor screening, consists of testing samples from several patients together leading to savings in costs and resources [26]. At the time this project was initiated, the TMA Procleix^®^ assay was the only SARS-CoV-2 test for which pooling had been approved in Spain [23]. The cost of pooling 8 samples together or “8-pooling” was estimated assuming that symptomatic patients would be tested individually, therefore the probability of re-testing was calculated based on the prevalence of hidden pre-symptomatic and asymptomatic patients [27]:

Probability of a pooling test being positive = 1 − (1 − λ)^ω^

where λ represents the proportion of hidden asymptomatic and pre-symptomatic in the population and ω the number of individual samples being pooled on each pooling test (ω = 8). Additionally, each person tested (either individually of pooled) would require a primary care visit for the sample extraction.

In order to estimate the cost of hospitalization, it was assumed that length of stay and ICU were 10.4 and 23 days, respectively [28,29,30,31]. For primary care costs, in addition to the cost for sample collection for testing, it was assumed that 50% of patients with mild symptoms would require a face-to-face visit, while the other 50% would have a consultation over the phone. Uncertainty around this assumption was also evaluated using one-way sensitivity analyses and results are presented in Table S4.

All unitary costs were updated to 2020 using the Consumer Price Index, when applicable, and are detailed in Table S5.

#### 2.3.2. Correlation between GDP Variation and NPI

The correlation between GDP change and NPI for each scenario was assessed through a multiple linear regression model based on data from 42 countries available on the OECD website [5]. The dependent variable was Q2 2020-vs. Q2 2019 GDP change, and the independent variables were the level of testing (positivity rate) [4], pandemic spread (case fatality rate) [4], economic structure (proxied by the share of manufacturing gross added value on total GDP) [32], and level of restrictions (Stringency Index) [3,4]. Initial correlation between the dependent variable and each of the independent variables is presented in Figure S3. The following multiple linear regression model was built:

Y = β_0_ + β_1_*Positivity rate + β_2_*Case fatality rate + β_3_*% industry over GDP + β_4_*Stringency Index

where Y represents the percentage difference on GDP from the same quarter of the previous year; β_0_ is the constant; β_1_ is the coefficient for the positivity rate; β_2_ is the coefficient for case fatality rate; β_3_ is the coefficient for the industry share over the GDP; and β4 is the coefficient for Stringency Index. The estimated values for these coefficients are presented in Table S4.

The out-of-sample fit of the econometric model was assessed using data for Q3 2020. The value estimated with our regression model was obtained based on the average testing rate (23.5 test/case) and Stringency Index (61.1) during July–September 2020. The estimated GDP was aligned with that recently reported for Q3 2020 in Spain [287,812 million € (M€) vs. 287,363 M€ corresponding to a 7.7% and 7.8% decline compared to Q3 2019, respectively].

To estimate Q4 2020 GDP for each scenario, Q4 2019 GDP was adjusted applying the estimated coefficients for Stringency Index and positivity rate obtained through the multiple linear regression model.

## 3. Results

### 3.1. Epidemiological Outcomes

With no changes in testing or stringency (scenario 1), the model estimated 2.38 million new exposed cases in the subsequent 90 days (Table 1), with a 2.1% error (calculated as the sum of square error between reported and modeled new confirmed cases, adjusted with e^−t^ function so that the recent error had a higher weight than the old one, divided by the number of cases error).

**Figure 3:**
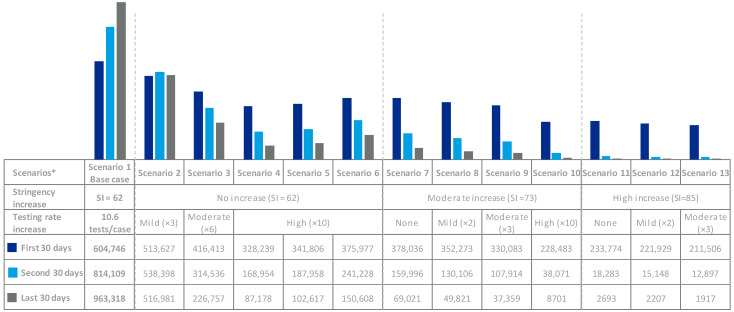
Exposed cases by 30-day period and scenario. * All scenarios consider a sensitivity of 96% except scenario 5 (89% sensitivity) and scenario 6 (73.3% sensitivity).

Both increased testing and stringency reduced cases, hospitalizations, and deaths. Scenario 4 (high testing rate increase with no stringency increase) and scenario 7 (moderate stringency increase with no testing rate increase) would lead to similar reductions of cases, hospitalizations, and deaths. A 3-fold testing rate increase with no stringency increase (scenario 2) would begin to bend the curve (Figure 3).

When looking into test sensitivity, scenario 5 (89% sensitivity) and 6 (73.3% sensitivity) would lead to 48,010 and 183,442 more cases than scenario 4 (96% sensitivity), respectively (Table 1).

### 3.2. Economic Outcomes

Scenarios with increased stringency (11–13) entailed the lowest total direct costs ranging from 315 M€-to-443 M€, whereas those with moderate (7–10) and no increase (1–6) led to higher direct costs, 586 M€-to-1303 M€ and 1784 M€-to-2993 M€, respectively, depending on the testing rate.

Scenarios 5 and 6, using less sensitive tests, resulted in 179 M€ and 682 M€ increases in total direct costs compared with scenario 4 (same testing rate and stringency, but higher sensitivity), respectively (Table 2).

**Figure 4:**
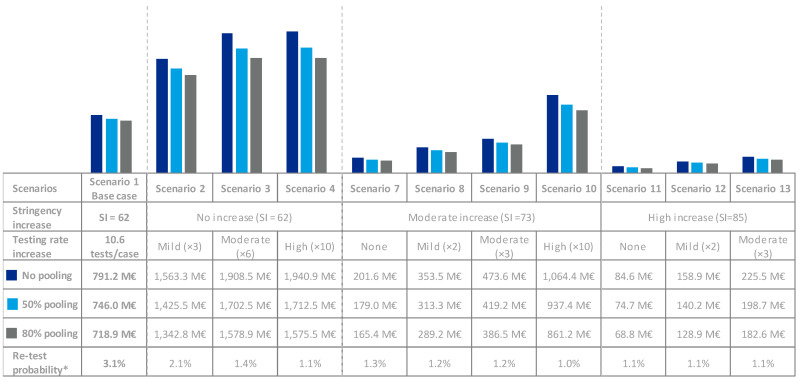
Pooling Costs * Re-test probability if pooling is implemented, calculated based on the prevalence of asymptomatic and pre-symptomatic individuals. M€: million €.

**Figure 5:**
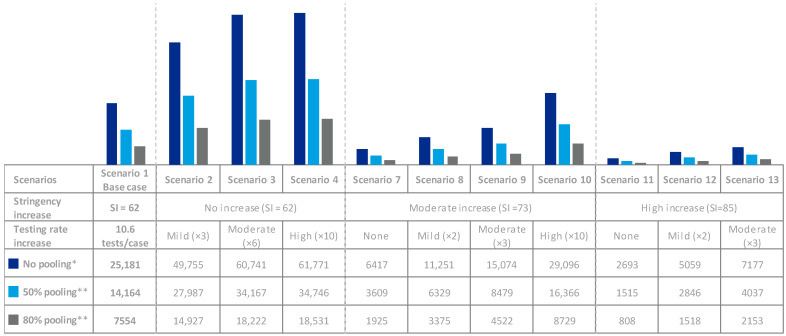
Pooling Efficiency. * Thousand tests and thousand individuals tested. ** Thousand tests.

For all scenarios, testing represented a relevant share of direct costs; however, these could be reduced using pooling techniques (Figure 4). Through all scenarios, performing 50% and 80% of tests via 8-pooling would reduce the testing costs between 11% and 17%, respectively. Additionally, fewer tests would be required to screen the same number of individuals (Figure 5). Implementing 50% and 80% of 8-pooling would almost double (×1.8) or more than triple (×3.3) testing capacity, respectively.

#### 3.2.1. Potential Impact of NPI on GDP

Results from the regression model indicated that lower positivity rates (higher testing rate), lower case fatality rate, higher GDP industry share, and lower Stringency Index correlated with lower GDP decline during Q2 2020 (Table S4 and Figure S3).

**Figure 6:**
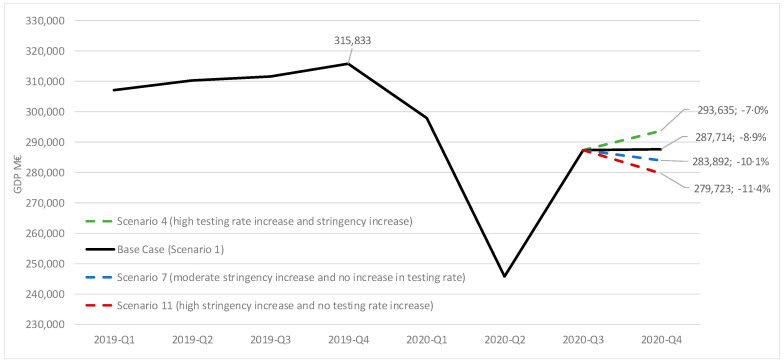
Potential Q4 GDP 2020 evolution for different scenarios of testing and stringency. GDP in million €; % change vs. GDP from Q4 2019. M€: million €.

When applying the coefficients for stringency and testing rates to the different scenarios, a difference of up to 4.4% points is observed for Q4 2020 GDP (Figure 6). For instance, scenario 4 (high testing rate increase with no stringency increase) is associated with a 8.9% drop in GDP, 1.9% points lower than our base case (scenario 1), whereas scenarios 7 and 11 (moderate and high stringency increase, respectively, with no testing rate increase) are associated with GDP drops of 10.1% and 11.4%, respectively, 1.2% and 2.5% points higher than our base case.

#### 3.2.2. Economic Value of Testing

**Figure 7:**
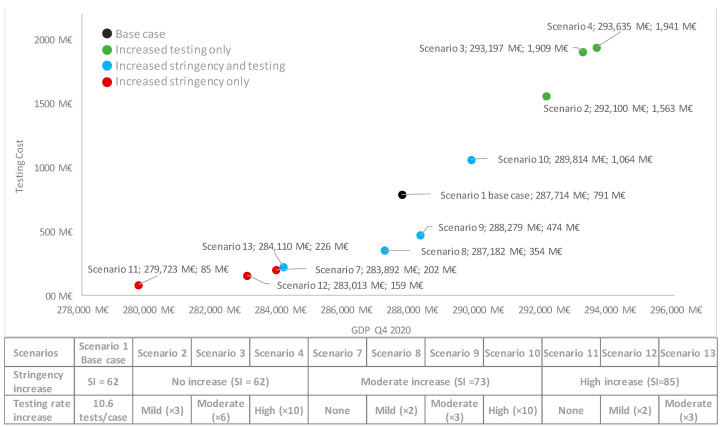
Costs of testing and GDP impact for each scenario. M€: million €; SI: Stringency Index.

Based on the correlations observed between lower positivity rates (higher testing rate), despite the high cost of testing, particularly for those scenarios which manage to bend the curve by increasing the testing rate (i.e., scenarios 3 and 4) a potential positive impact on GDP is observed of Q4 2020. Additionally, when comparing scenarios 4 and 7, leading to similar health outcomes (Table 1 and Figure 4) although reflecting very different policies (high testing rate increase vs. moderate stringency increase), in spite of the increased testing costs for scenario 4, this seems to be associated with a positive impact on GDP vs. scenario 7 (Figure 7).

## 4. Discussion

The decentralized system that governs health policy decisions in Europe has led to each country, and even each region within some countries, implementing different NPI to face the COVID-19 pandemic [3,4,33]. This hinders the definition of a single scenario that may be representative for Europe, and even for Spain, as a whole. In spite of the regional diversity, we assume the scenario that best represents the situation in Spain during Q4 2020 would be close to scenario 7 (moderate stringency increase with no testing rate increase) [3,4]. Other European countries like Austria, Italy, France and Germany implemented stricter lockdowns during Q4 2020, closer to scenario 11 (high stringency increase), whereas Belgium or the UK showed lower levels of restrictions for most of Q4 2020, close to our base case, and even lower for some of the Nordic countries [3]. Testing rates are also variable across countries ranging from <3 to >80 cases/positive, Iceland and Denmark being the countries with the highest testing rates and with strategies that would be close to scenario 4 (high testing rate increase with no stringency increase) [3,4]. Whether these measures will be sufficient to fully control the spread of COVID-19 before high vaccination rates are reached and the macroeconomic impact these may entail is still unknown. However, according to the correlations observed through our regression model for Q4 2020, some countries fall into scenarios that may entail negative macroeconomic impacts (like scenario 7 and 11) when compared to others with less stringency and/or increased testing rates (i.e., scenarios 2–4 or 8–10).

The SEIR epidemiological model shows that an increased testing rate in September 2020, without any further restrictions, would have reduced the rate of new cases in Spain. Scenario 2 (mild testing rate increase) already illustrates a curve that is starting to bend (Figure 3). Further increase in the testing rate could lead to reductions in the number of cases in line with that observed when stringency is increased. For instance, when comparing scenarios 4 (high testing increase) and 7 (moderate stringency increase), scenario 4 results in slightly fewer cases, hospitalizations and deaths (and associated costs) at the expense of higher testing costs, but overall, resulting in a positive impact of 9743M€ in GDP for the Spanish case. All scenarios relying on increased testing strategies show positive correlations with GDP; whereas the opposite trend is observed for those with increased stringency.

Testing costs represent an important share of direct costs, particularly when used in massive screening approaches. Provided the selected test is adequately sensitive and is used for screening low prevalence groups (i.e., to identify asymptomatic and pre-symptomatic individuals in an effort to keep re-testing probabilities minimized), testing costs may be mitigated through the implementation of pooling strategies [23]. Additionally, pooling techniques may also prove efficient to address the need for increased throughput capacity.

When considering sensitivity, differences of over ×3000 in LoD have been observed within molecular tests [13,14], and when comparing different techniques, like the antigen test [34], not initially considered in this project as it was only recommended for symptomatic patients by the Spanish Authorities at a later stage. Our model shows that reducing sensitivity, from 96% to 89% and 73.3% [24,25], led to increases of 8.2% and 31.4% in cases, and 5.1% and 19.2% in hospitalizations, respectively. It is therefore critical to choose the appropriate test in terms of sensitivity for the particular target population (i.e., symptomatic, asymptomatic), when considering the impact of screening strategies on healthcare systems. It is worth mentioning that test specificity was not assessed within this project given that most tests have shown very high levels of specificity and therefore the global impact of isolating potential false positives for 14 days is expected to be minimal [13].

Although comparison with prior work is challenging given the lack of previous studies estimating the impact of NPI on GDP in Spain and due to the different approaches used for estimating the GDP decline and/or scenarios considered, our results are aligned with those from previous models. For instance, previous research based on a Susceptible-Infected-Recovered (SIR) model in the US, estimated that a general lockdown of the population would lead to a 37.7% drop in GDP. This could be reduced to 24.9% if lockdown was more stringent for the older population than for the younger active population and to 7% if social distancing and a testing, tracing, and isolation (TTI) strategy was also implemented [16]. A similar study, also using a SIR model, estimated an improvement in GDP of 6% if 5% of the US population were tested every week [17]. A modified SEIR model estimating the economic impact of different interventions in the UK concluded that implementation of population-scale TTI strategies, could reduce the GDP decline associated with additional lockdowns in half—from £1180 billion to £503 billion [18].

Spain was selected to assess the impact of the different NPI on the clinical and economic outcomes of the pandemic because, together with Italy, these were the first countries to be hit by the COVID-19 pandemic in Europe. At the end of the first COVID-19 wave (when this project was initiated), Spain was one of the European countries with the highest number of cases, hospitalizations and deaths and also one of the countries that implemented the strictest lockdowns in Europe, which overall presented a suitable scenario to evaluate the potential clinical and economic impact of the different strategies against the COVID-19 pandemic. However, the fact that the epidemiological model is based on Spanish data may only prevent our results from being fully and directly extrapolated to other regions.

The number of confirmed cases estimated by the epidemiological model for the first 30 days was actually reached after 37 days, indicating a relatively accurate predictive capacity. This delay in reaching the confirmed number of cases may be explained by additional restrictions applied in some regions, or the slight reduction in testing rates (8.3 tests/positive the week prior to 19 October 2020) compared to the one considered in our base case scenario (10.6 tests/positive) [2,4]. Additionally, the number of exposed cases (2.38 million) would be in line with the figures reported from the first wave, at the end of which a seroprevalence of 5.0% was reported for the Spanish population (47.3 million) [20]. A recent update of the seroprevalence study conducted in Spain, reported a preliminary seroprevalence of 9.9% in November 2020, therefore indicating that over 2.5 million people may have seroconverted between since June 2020, also in line with our estimates [35].

Regarding the economic model, it is worth mentioning that the regression model was developed using data from 42 countries on COVID-19 spread, economic structure, NPI, and GDP change during Q2 2020. While our model does not aim to replace those more oriented to GDP forecasting such us general equilibrium models, and while reports on Q4 2020 GDP are still pending, this approach may allow exploring the correlation between NPI and GDP variation by other countries in their efforts to predict macroeconomic COVID-19 impacts. When used for assessing the potential impact of NPI on Q3 2020 GDP in Spain, the model demonstrated excellent accuracy (i.e., 287,812M€ vs. 287,363M€ corresponding to a 7.7% and 7.8% decline relevant to Q3 2019, respectively). Although the pursued objectives and the methodological approaches are different, the impact of the different restrictions is aligned with those predicted by the Bank of Spain (BoS) [36]. The BoS forecasts a GDP decline in Q4 2020 of ~7.0% and ~12.0% compared to Q4 2019, for scenarios with lenient and stringent restrictions, respectively, whereas our model estimates a decline between 7.0% (scenario 4) and 11.4% (scenario 11). Although the scenarios considered by the BoS are not fully aligned with the ones considered herein, differences in the quarter-to-quarter GDP decline are aligned.

Previous works have aimed to correlate epidemiological and macroeconomic variables, for instance the October 2020 International Monetary Fund (IMF) Report includes a model correlating the level of lockdown measured through the Stringency Index with the GDP drop and also considers the severity of the pandemic through the COVID-1 cases per capita [37]. Differently to our model, the IMF one does not investigate the correlation between testing and GDP drop. However, a recent report from the United Nations Conference of Trade and Development highlighted the necessity of testing facilities to ensure a safe return to work and business [38], therefore increased testing may facilitate reopening economical activities.

Both the epidemiological and economic models developed herein exhibit strengths and limitations. Our SEIR model predicted a much higher rate of hospitalizations, and deaths than those actually observed. This may be explained by the fact that data used to inform the model primarily corresponds to the first wave; different in nature to the second wave. For instance, testing capacity is now higher, healthcare professionals have more experience and tools to manage COVID-19; the use of a mask is now mandatory, and the age distribution of COVID-19 patients seems to have shifted towards a younger population [22,39]; and although some behavioral changes have been indirectly considered because the model prediction has been fitted to the actual new confirmed cases, this has not been reflected in the estimated hospitalization rate. Additionally, the national-level epidemiological data does not have adequate granularity to fully describe the dynamics of COVID-19 (i.e., new daily cases and hospitalizations stratified by age and sex is not currently publicly available) [40]. However, the overestimated hospitalization rate has been overcome by adjusting it to 5.8% in line with that reported by the Spanish Authorities for the first 37 days of the model’s prediction, which led to a mortality rate of 1.1%. The impact of this variable has been further explored through a one-way sensitivity analysis by applying a 20% increase and decrease to the hospitalization rate; differences of over 300M€ may be expected should future COVID-19 waves entail lower or higher rates of hospitalization. Another limitation of the epidemiological model is that confirmed patients have been assumed to be well isolated and therefore, not able to transmit the infection. Different levels of adherence to isolation have not been explored due to the lack of evidence on isolation compliance and its consequences. Another limitation of the scenarios explored herein is that NPI are applied to the whole population and therefore, alternative options of addressing the highest risk separately from the overall population have not been considered. Additionally, our model also considers that no re-infection is possible. While rates of confirmed reinfection are still low, current evidence suggests that immunity lasts for a minimum of 4–7 months, and therefore the impact on our 90-days estimate should be minimal [41,42,43,44,45]. Additionally, the impact of immunity due to vaccination has not been considered either. With the exception of Israel, the United States, the United Arab Emirates, the United Kingdom, and Chile, vaccination rates are still very low (<10%) across most countries [4]; also, even though recent evidence seems to suggest that vaccination immunity should be as effective as natural immunity, this is still not fully understood [45]. Future updates to our model when further evidence is available may be able to address some of these outstanding questions.

The economic models developed herein also entail some limitations. For instance, the fact that the correlation between NPI and GDP drop has been assessed using data from countries with very different economies and in different cyclical positions at the beginning of the pandemic. Additionally, nominal GDP was used for both the regression model and the subsequent economic impact for each scenario. Another limitation is that test sensitivity could not be included in the regression model and therefore, only differences in direct costs can be estimated when exploring this parameter. It is worth mentioning that some costs have not been accounted for, such as emergency room visits, hospitalizations at home, subsequent visits to primary care, cost of contact tracing, lockdown-related mental health costs, or its longer-term effects such as depression or suicides.

## 5. Conclusions

In spite of the increased availability of vaccines, herd immunity is not expected to be reached before the end of 2021 [46]. In the meantime, despite the limitations highlighted above, these models may be useful tools for health policy makers during current and future waves of COVID-19, should they occur under similar conditions to support informed decision-making, so that the population is placed in a future that balances both health and socioeconomic concerns.

## Figures and Tables

**Table 1:** Total number of new exposed cases, hospitalizations and deaths estimated by scenario.

Scenario *	Stringency Increase	Testing Rate Increase	Exposed Cases	Hospitalizations	Deaths
Scenario 1: Base case	SI = 62	10.6 tests/case *	2,382,172	97,488	18,676
Scenario 2	No increase SI = 62	Mild (×3) *	1,569,006	72,111	15,730
Scenario 3	No increase SI = 62	Moderate (×6) *	957,706	51,212	13,069
Scenario 4	No increase SI = 62	High (×10) *	584,371	37,099	11,058
Scenario 5	No increase SI = 62	High (×10) **	632,381	38,996	11,343
Scenario 6	No increase SI = 62	High (×10) ***	767,814	44,206	12,100
Scenario 7	Moderate increase SI = 73	None *	607,053	38,502	11,440
Scenario 8	Moderate increase SI = 73	Mild (×2) *	532,199	35,450	10,964
Scenario 9	Moderate increase SI = 73	Moderate (×3) *	475,356	33,066	10,577
Scenario 10	Moderate increase SI = 73	High (×10) *	275,255	24,230	9005
Scenario 11	High increase SI = 85	None *	254,751	23,398	8902
Scenario 12	High increase SI = 85	Mild (×2) *	239,284	22,674	8757
Scenario 13	High increase SI = 85	Moderate (×3) *	226,320	22,064	8631

SI: Stringency Index. * All scenarios except 5 and 6 consider a level of sensitivity of 96%. ** 89% sensitivity. *** 73.3% sensitivity.

**Table 2:** Direct costs by type of cost for each scenario.

Scenario *	Stringency Increase	Testing Rate Increase	Hospitalization	ICU	Primary Care	Individual Testing	Total
Scenario 1: Base case	SI = 62	10.6 tests/case *	504.3 M€	347.6 M€	140.7 M€	791.2 M€	1783.7 M€
Scenario 2	No increase SI = 62	Mild (×3) *	373.0 M€	257.1 M€	101.6 M€	1563.3 M€	2295.1 M€
Scenario 3	No increase SI = 62	Moderate (×6) *	264.9 M€	182.6 M€	67.6 M€	1908.5 M€	2423.6 M€
Scenario 4	No increase SI = 62	High (×10) *	191.9 M€	132.3 M€	45.2 M€	1940.9 M€	2310.3 M€
Scenario 5	No increase SI = 62	High (×10) **	201.7 M€	139.0 M€	48.2 M€	2100.3 M€	2489.3 M€
Scenario 6	No increase SI = 62	High (×10) ***	228.7 M€	157.6 M€	56.4 M€	2550.1 M€	2992.8 M€
Scenario 7	Moderate increase SI = 73	None *	199.2 M€	137.3 M€	47.5 M€	201.6 M€	585.6 M€
Scenario 8	Moderate increase SI = 73	Mild (×2) *	183.4 M€	126.4 M€	43.9 M€	353.5 M€	707.2 M€
Scenario 9	Moderate increase SI = 73	Moderate (×3) *	171.0 M€	117.9 M€	40.6 M€	473.6 M€	803.2 M€
Scenario 10	Moderate increase SI = 73	High (×10) *	125.3 M€	86.4 M€	27.1 M€	1064.4 M€	1303.3 M€
Scenario 11	High increase SI = 85	None *	121.0 M€	83.4 M€	26.1 M€	84.6 M€	315.2 M€
Scenario 12	High increase SI = 85	Mild (×2) *	117.3 M€	80.8 M€	25.6 M€	158.9 M€	382.7 M€
Scenario 13	High increase SI = 85	Moderate (×3) *	114.1 M€	78.7 M€	24.9 M€	225.5 M€	443.2 M€

ICU: Intensive Care Unit; SI: Stringency Index. * All scenarios except 5 and 6 consider a level of sensitivity of 96%. ** 89% sensitivity. *** 73.3% sensitivity.

## Data Availability

Publicly available datasets were analyzed in this study and can be found here: daily diagnosed cases in Spain: https://cnecovid.isciii.es/covid19/#documentación-y-datos (accessed on 27 October 2020); testing rate per country https://ourworldindata.org/coronavirus-testing (accessed on 27 October 2020); COVID-19 case-fatality rate per country: https://ourworldindata.org/grapher/COVID-cfr-exemplars (accessed on 11 September 2020); stringency index per country: https://ourworldindata.org/grapher/COVID-stringency-index (accessed on 27 October 2020); Q2 2020 GDP change and GDP per capita: https://data.oecd.org/gdp/gross-domestic-product-gdp.htm (accessed on 27 October 2020) GDP industry share per country: https://data.worldbank.org/indicator/NV.IND.TOTL.ZS?view=chart (accessed on 27 October 2020). Full study data is available at the following link: https://doi.org/10.34810/data111.

## Supplementary Materials

The following are available online at https://www.mdpi.com/article/10.3390/v13050917/s1, Figure S1: Patient flow between epidemiological compartments, Figure S2: Contact matrix adjusted by age group, Figure S3: Correlation between epidemiological variables and economic impact, Table S1: Epidemiological model parameters and characterization, Table S2: Parameters adjusted by age group, Table S3: One-way sensitivity analysis of hospitalization rate, Table S4: One-way sensitivity analysis of face-to-face primary care visits, Table S5: Unitary costs, Table S6: Estimated coefficients of the regression model.
