# Supplementary material for: Social Restrictions versus Testing Campaigns in the COVID-19 Crisis: A Predictive Model Based on the Spanish Case
Source: Viruses. 2021 May 15;13(5):917. doi: 10.3390/v13050917 (PMC8157049; doi:10.3390/v13050917)
Supplement: Supplementary file 1 [file viruses-13-00917-s001.zip › viruses-1174874-supplementary.pdf]

Social restrictions versus testing campaigns in the COVID-19 crisis: a predictive model based on the Spanish case. Supplementary material

Content:

Figure S1: Patient flow between epidemiological compartments
Figure S2: Contact matrix adjusted by age group
Figure S3: Correlation between epidemiological variables and economic impact
Table S1: Epidemiological model parameters and characterization
Table S2: Parameters adjusted by age group
Table S3: One-way sensitivity analysis of hospitalization rate
Table S4: One-way sensitivity analysis of face-to-face primary care visits
Table S5: Unitary costs
Table S6: Estimated coefficients of the regression model.

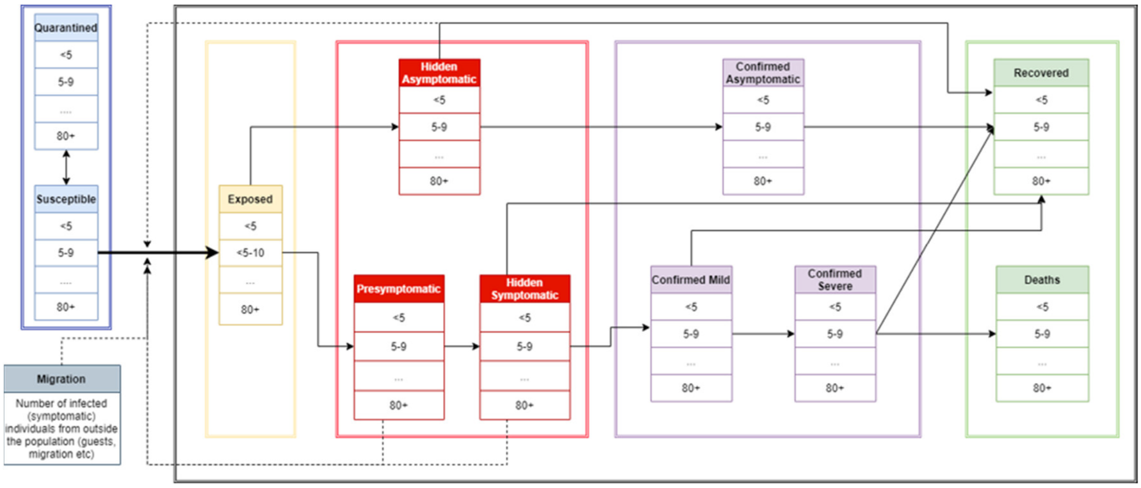

Figure S1. Patient flow between epidemiological compartments

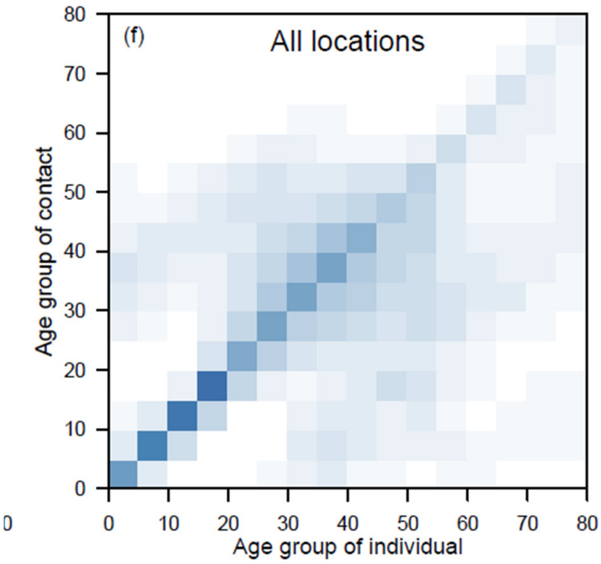

Figure S2. Contact matrix adjusted by age group

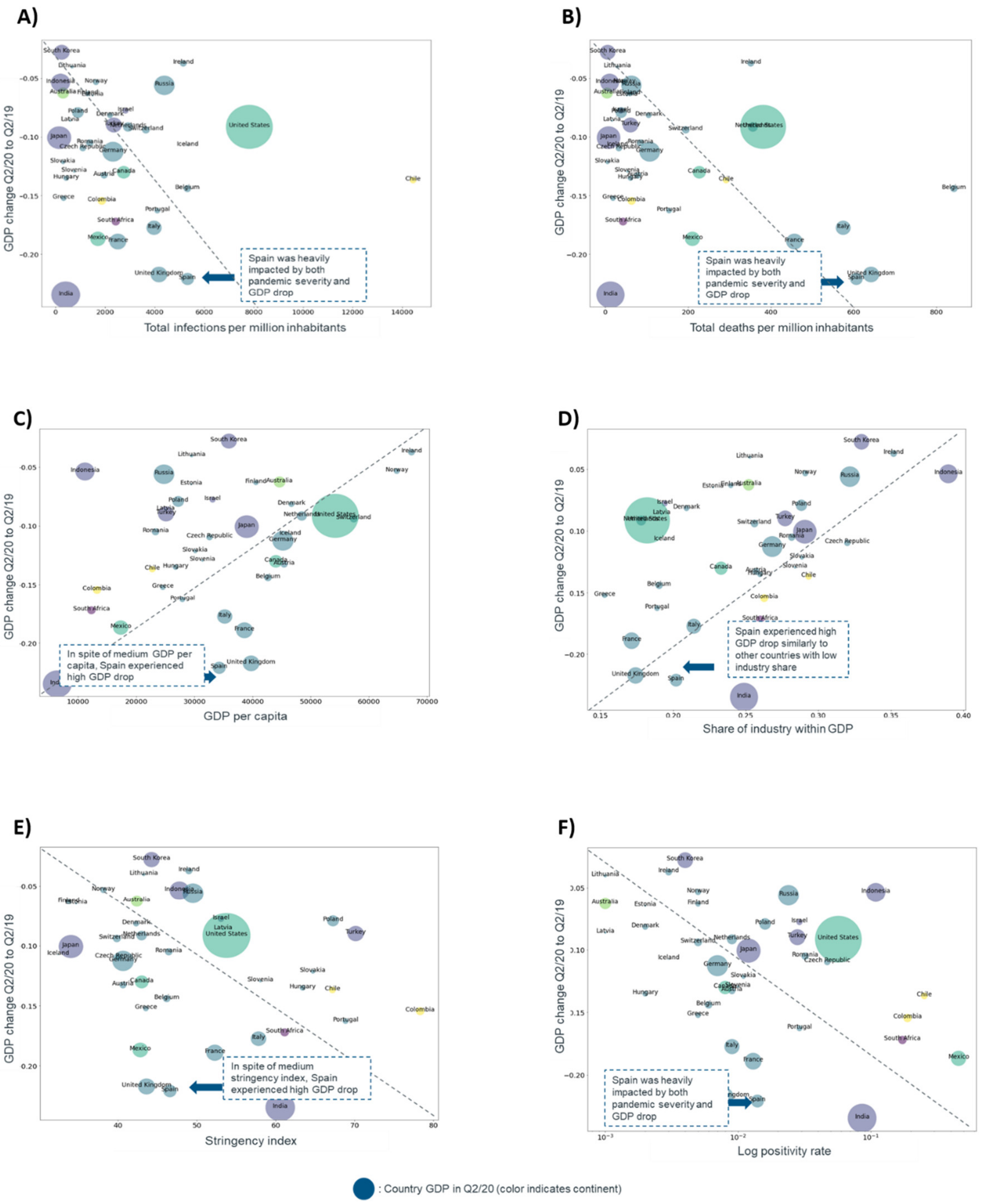

Figure S3. Correlation between epidemiological variables and economic impact

Note: A) Higher pandemic severity in terms of total infections per million inhabitants is related to higher GDP drop; B) Higher pandemic severity in terms of total deaths per million inhabitants is related to higher GDP drop; C) Higher GDP per capita is related to lower GDP drop; D) Higher share of industry is related to lower GDP drop; E) Higher stringency index (lockdown policy), is related to higher GDP drop.

Table S1. Epidemiological model parameters and characterization

Compartment		Definition	Key parameter (transition rate)			Rationale from transition to next compartment
			Parameter	Value	Source	
**Susceptible**		Individuals who are potential to contract the disease if they come in contact with an infected individual	*β_a:* probability of transmission during single contact with asymptomatic person	0.002	Fitted to data, from range [0.0001, 0.05]	We estimate average number of contacts per day starting with Contact Matrix, then reduce it based on Stringency Index<br>Afterwards, we multiply the number of contacts by size of hidden group and beta to determine the newly exposed
			β_pre: probability of transmission during a single contact with pre-symptomatic person	0.186	Fitted to data from range [0.01, 0.2]	
			β_s: probability of transmission during a single contact with symptomatic person	0.01	Fitted to data from range [0.01, 0.2]	
			Age immunity: adjustment to beta based on age of susceptible individual		Constant adjustment estimated to have correct age group promotion in new cases	
			Season_adjustment: adjustment to beta based on season; bigger transition on winter	-0.23	Fitted to data from range [-1; 1]	
**Exposed**		Individuals who are in the incubation phase. They will return negative if tested, and are not transmitting the virus	*E->H:* constant share of exposed who transition to the hidden stage<br>*Asymptomatic_by_age:* probability of developing the symptoms by age	0.18	Fitted to data from range [0.15; 0.6]	After 1/E->H days (on average) from infection, the individual finishes incubation. Probability of being symptomatic depends upon age
**Hidden**	**Hidden Asymptomatic**	Individuals who are infected, not detected and will never develop any symptoms. They will return positive if tested, and are transmitting the virus	*H_A->R:* constant share of asymptomatic is moved to recovered	0.077	Fitted to data from range [0.07, 0.5]	We estimate testing pool size (the faster development spread, the bigger), then randomly choose who from that pool would be tested<br>If the individual is not confirmed after 1/H_A->R number of days it would become recovered
			Testing_pool_size: estimation of people that are target to be tested. Depends on new confirmed group from previous day	21	Number of contacts of new confirmed (NC)*pool_modifier	
			Test_number: absolute number of tests executed each day		Historical: based on data<br>Modelling: estimated based on provided tests per case value by user	
			Test sensitivity: proportion of correctly identified positives	0.96	Historical: based on test ratio<br>Modelling: provided by user	
	**Hidden Pre-symptomatic**	Individuals who are infected, not detected, have not developed any symptoms, but will do it in future. They are transmitting the virus with higher rate than asymptomatic	*H_pre->H_S:* constant share of pre-symptomatic is moved to symptomatic<br>testing_pool_size, test_numer, test sensitivity	0.363	Fitted to data from range [0.2, 1]	We estimate testing pool size (the faster development spread, the bigger), then randomly choose who from that pool would be tested<br>If the individual is not confirmed after 1-2 days, it would become symptomaticsymptomatic

	**Hidden Symptomatic**	Individuals who are infected, not detected and have developed symptoms. They are transmitting the virus with a slightly smaller rate than pre-symptomatic	*H_S->C_M:* constant share of asymptomatic is moved to confirmed testing_pool_size, test_numer, test sensitivity	0.329	Fitted to data from range [0.1, 1]	Certain share of symptomatic would be diagnosed each day (even without testing)
**Confirmed**	**Asymptomatic**	Asymptomatic individuals who were tested positively and are isolated. We assume that isolation is strict, so they are no longer spreading	*C_A->R:* constant share of asymptomatic confirmed are moved into recovered	0.07	Constant based on quarantine time	After quarantine is over, individuals are moved into recovered
	**Mild**	Individuals confirmed with COVID-19, who are symptomatic or are about to be symptomatic, but do not need hospitalization. They are isolated and not spreading	*C_M->R*:* constant share of confirmed mild is moved into recovered	0.07	Constant based on quarantine time	Confirmed mild eventually get better and get removed, or get worse and are hospitalized
			C_M->C_S:* constant share of confirmed mild is moved into severe	0.037	Constant based on hospitalization vs confirmed rate	
	**Severe**	Individuals confirmed with COVID-19 who are hospitalized. They are not spreading	*C_S->R*:* constant share of confirmed that are released	0.02	Constant based on average time from hospitalization to fully recovered.	When individual leaves the hospital or passes away, they are moved to removed group
			C_S->D:* constant share of population that doesn't survive.	0.009	Constant based on total death to hospitalization ratio	
**Removed**	**Recovered**	Individuals who are done with the infection and are no longer quarantined. They developed resistance they will be tested negatively	-		-	-
	**Death**	Individuals who didn't survive the infection	-		-	-

Table S2. Parameters adjusted by age group

Age group	% of asymptomatic	Sensitivity adjustment
0 to 4	0.9	0.1
5 to 9	0.9	0.1
10 to 14	0.83	0.1
15 to 19	0.76	0.2
20 to 24	0.69	0.33
25 to 29	0.62	0.4
30 to 34	0.55	0.5
35 to 39	0.48	0.6
40 to 44	0.41	0.7
45 to 49	0.34	0.8
50 to 54	0.27	0.9
55 to 59	0.2	1.0
60 to 64	0.2	1.1
65 to 69	0.2	1.2
70 to 74	0.2	1.3
75 to 79	0.2	1.4
80+	0.2	1.5

Table S3. One-way sensitivity analysis of hospitalization rate

Change	% hosp.	Epidemiological outputs			Economic outputs		
		Hosp.	ICU	Deaths	Hosp.	ICU	Total costs
**-20% Δ**	4,64%	70.933	7.058	14.941	403,4 M€	278,1 M€	1.613,4 M€
**= Δ**	5,80%	88.666	8.822	18.676	504,3 M€	347,6 M€	1.783,7 M€
**+20% Δ**	6,96%	106.399	10.586	22.411	605,2 M€	417,1 M€	1.954,1 M€

Note: only showing parameters affected by a change on hospitalization rate. All other outputs remain the same as the base case.

Table S4. One-way sensitivity analysis of face-to-face primary care visits

Change	% face-to-face visits	Economic outputs	
		PC	Total costs
**-20% Δ**	40,0%	130,9 M€	1.773,9 M€
**= Δ**	50,0%	140,7 M€	1.783,7 M€
**+20% Δ**	60,0%	150,5 M€	1.793,6 M€

Note 1: non-face-to-face primary care visits were computed as consultations over the phone.

Note 2: only parameters affected by a change on face-to-face primary care visits rate are displayed.

Table S5. Unitary costs

Input	Unit cost (€)	Source
Individual molecular test assay	18.00	Grifols data on file
8-pooling TMA test assay	10.80	
Total individual molecular test (including logistics, healthcare professional and other costs)	31.42	
Total 8-pooling TMA test (including logistics, healthcare professional and other costs)	21.88	
Hospitalization, per day	546.88	MSCBS [1]
Intensive Care Unit hospitalization, per day	1,713.00	eSalud [2]
Telephone General Practitioner visit	28.00	eSalud [2]
Face to Face General Practitioner visit	58.00	eSalud [2]

1. Ministerio de Sanidad Consumo y Bienestar Social (MSCBS). Portal Estadístico. Registro de Atención Especializada (RAE-CMBD) [Internet] https://pestadistico.inteligenciadegestion.mscbs.es/publicoSNS/Comun/ArbolNodos.aspx?idNodo=23606
2. Consulting O. eSalud - Información económica del sector sanitario. 2020 [Internet] http://esalud.oblikue.com/

Table S6. Estimated coefficients of the regression model

Dependent variable	R-squared	F-statistic	Condition number
GDP change	0.533	9.986	1,370

Variable	Coefficient	Standard error	P-value	VIF
Positivity rate	-0.2202	-0.3653	0.006	1.33
Case fatality rate	-0.4301	-0.3780	0.004	2.40
Industry share over total GDP	0.3766	0.3899	0.005	13.19
Stringency Index	-0.0011	-0.2338	0.059	11.74
